# Among the Colleges

**Published:** 1903-04

**Authors:** 


### EDITORIALS.

### AMONG THE COLLEGES.

THE announcement comes to us of the probable closing of the Veterinary Department of McGill University for want of adequate pecuniary aid. This, the second closing of Eastern institutions, comes as a serious blow to veterinary educational progress in America. It is peculiarly distressing and painful to those who have, in season and out of season, urged higher veterinary education. The work at McGill—formerly the Montreal Veterinary College—has ever been under the direction and care of Professor D. McEachran, and the work of that institution will ever remain a monument of honor and esteem to his unselfish devotion for the advancement of his chosen vocation. In times of depression and adversity, in the periods of the multiplication of mushroom veterinary colleges, from a thoroughly scientific course graded over a period of three years, this institution never wavered from its high sense of responsibility as an educational institution. When its income from student fees was meagre and insufficient for its maintenance the willing hand and open purse of its chief director carried it along and schooled its matriculants in the same manner as in other years, and it can be well and truly said that every graduate from that institution bears generous testimony to the good work so well done. In conclusion, may we not properly ask Canada, with her enormous live-stock wealth and industries, not to see this institution close for want of financial support? What will the province of Manitoba do for future additions to the profession, in that no other college graduates of Canadian institutions are eligible to practice in her Province?

